# Clinical and psychosocial characteristics of children with nonepileptic seizures

**DOI:** 10.4103/0972-2327.42935

**Published:** 2008

**Authors:** Sri Sankar Chinta, Prahbhjot Malhi, Pratibha Singhi, Sudesh Prabhakar

**Affiliations:** Department of Pediatrics, Post Graduate Institute of Medical Education and Research, Chandigarh, India; 1 Department of Neurology, Post Graduate Institute of Medical Education and Research, Chandigarh, India

**Keywords:** Clinical features, nonepileptic seizures, psychosocial stress, short-term outcome

## Abstract

**Objective:**

The aim of this study is to present a comprehensive profile of clinical and psychosocial characteristics of children with psychogenic nonepileptic seizures and to assess the short-term outcome of these patients.

**Materials and Methods:**

The subjects were consecutive cases of children with a diagnosis of nonepileptic seizures (N=17, mean age = 10.7 years, S.D. = 1.26) and two groups of control groups matched on age and sex: true seizure group and healthy controls. All the children were recruited from the out-patient services of the Department of Pediatrics of a tertiary care teaching hospital in North India. Detailed history taking and clinical examination was done in the case of every child. A standard 18 channel EEG was done in all the children and a video EEG was done in 12 cases of children with nonepileptic seizures. The Childhood Psychopathology Measurement Schedule (CPMS) and Life Events Scale for Indian Children (LESIC) were used to measure the children's emotional and behavioral functioning at home, and the number of life events and the stress associated with these events in the preceding year and the year before that. Short-term outcome was examined three to six months after the diagnosis of nonepileptic seizures was made.

**Results:**

Unresponsiveness without marked motor manifestations was the most common “ictal” characteristic of the nonepileptic seizures. Pelvic thrusting, upper and lower limb movements, head movements, and vocalization were observed in less than one-third of the patients. Increased psychosocial stress and significantly higher number of life events in the preceding year were found to characterize children with nonepileptic seizures, as compared to the two control groups. The nonepileptic seizures and true seizures groups had a higher proportion of children with psychopathology scores in the clinically significant maladjustment range, as compared to those in the healthy control group. A majority of the patients (82.4%) either recovered completely or had more than 50% reduction in the frequency of their symptoms, after three to six months of initiation of therapy.

**Conclusions:**

Psychosocial stress is common among children with nonepileptic seizures. Confirmatory diagnosis by video EEG, along with prompt psychosocial intervention, often results in a favorable outcome for most children with nonepileptic seizures.

## Introduction

Conversion disorder is a loss or alternation in sensory or voluntary function, that cannot be fully explained by known patho-physiological mechanism.[1] One of the common presentations of conversion disorder are “pseudo seizures” also called “psychogenic", “nonepileptic” or “hysterical” seizures. Nonepileptic seizures are clinical events that resemble epileptic seizures but are not associated with abnormal cortical electrical discharges.[2,3] Nonepileptic seizures are postulated to be the result of an unconscious psychological conflict, which is converted into symbolic somatic symptoms that reduce anxiety and protect the conscious self from stressful emotions. The symptoms also provide considerable secondary gain to the child, as the sick role generates attention and sympathy. It also allows the child to minimize personal responsibility for any failure and helps maintain its self-esteem.[4,5]

Nonepileptic seizures among children are relatively rare in the developed countries,[6] but several epidemiological and clinical studies have found nonepileptic seizures to be fairly common in the developing countries, including India.[7,8]

Since the advent of enhanced diagnosis by video EEG monitoring, nonepileptic seizures have become the focus of clinical and research attention.[9,10] Most of the studies have focused on the clinical distinctions between epileptic and nonepileptic seizures; few have focused on the underlying psychological antecedent factors or outcome of pseudo seizures in children. Hence, the current literature provides limited information about the clinical and psychosocial characteristics of these children and their families.

The present study therefore seeks to -

- present a comprehensive clinical profile of children with nonepileptic seizures, who were rigorously diagnosed and who did not have concomitant true epilepsy;
- study the number of life events and the associated stress related to these events;
- study the psychopathology among children with nonepileptic seizures and contrast it with control groups;
- assess the short-term outcome among children with nonepileptic seizures.

## Materials and Methods

### Sample

All consecutive children in the age range of six to 14 years and with a diagnosis of nonepileptic seizures, presenting to the Department of Pediatrics' outpatient services of a tertiary care teaching hospital in North India, were enrolled. The diagnosis of nonepileptic seizures was made on the basis of clinical history, direct observation, clinical examination, EEG, neuro imaging, and, in some cases, video EEG.

For inclusion, children had to meet the following criteria:

- compliance with the diagnostic criteria for nonepileptic seizures^1^
- no other clinical evidence suggesting that the seizures were due to epilepsy or any other neurological disorder.

Children were excluded if they had any known neurological disorder, static or progressive, history of chronic systemic illness, or grade III or IV protein energy malnutrition. In all, 30 children were evaluated over a period of one year (January 2003 to December 2003). Out of these, 17 children met the inclusion criteria and were enrolled in the study.

These children were matched on the basis of age and sex to two control groups: seizure control group and healthy controls. The International League against Epilepsy (ILAE) diagnostic criteria were used for the diagnosis of epilepsy. The seizure control group was enrolled from the Neuro Cysticercosis Clinic and Neuro Developmental Clinic from the Department of Pediatrics of the same hospital. Children of the hospital employees working in the Department of Pediatrics were enrolled as healthy controls group after matching for age and sex.

All the children had a follow-up for a period of three to six months and the short-term outcome was noted after the end of three to six months.

### Instrumentation

Children with nonepileptic seizures were compared to two matched groups of children, healthy controls and true seizures group, on two parent reported measures: Childhood Psychopathology Measurement Schedule (CPMS) and Life Events Scale for Indian Children (LESIC).

- *Childhood Psychopathology Measurement Schedule*: Emotional and behavioral functioning at home was measured by the mother's rating on the Childhood Psychopathology Measurement Schedule (CPMS),[11] which is the Indian adaptation of the Child Behavior Checklist[12] (CBCL), one of the most widely used instruments measuring emotional and behavioral problems in children. The CPMS consists of 75 problem items on which parents rate their child using a three point scale, with higher scores reflecting more problems. The CPMS has eight sub scales, including low intelligence and behavior problems, anxiety, depression, conduct problems, somatization, and special symptoms. The authors also recommend a cut off score of 10 and children scoring 10 and above are considered as exhibiting clinically significant level of maladjustment.

- *Life Events Scale for Indian Children:* The Life Events Scale for Indian Children[13] (LESIC) was administered to all the children, to study the number of life events that the child had experienced and the stress associated with these events. The LESIC is a bilingual (Hindi and English) scale which comprises 50 life events, hierarchically arranged in increasing order of stress. The parents are asked whether the events listed in the scale occurred in the preceding year or the one prior to the preceding year. The stress scores for the events that had occurred were added to yield a total stress score. The scale yields four scores: number of life events in the last one year and the preceding year and the total stress score in the last one year and the preceding year.

### Statistical analysis

The clinical presentation of the pseudo seizure group was studied by calculating the percentages for the various symptoms present and comparing the results of our study to previous studies. Group comparisons were done using the chi square test.

## Results

Among the 17 children diagnosed with nonepileptic seizures, 13 (76.5%) were girls and four (23.5%) were boys. The mean age of the pseudo seizure group was 10.7 years, with an age range of 7-13 years. The sample was predominantly female and the male: female ratio was 1 : 3.25.

### Clinical characteristics of nonepileptic seizure group

Analysis of the clinical characteristics of the nonepileptic seizure group revealed that upper limb movements were observed in 12 children (70.6%), asymmetrical clonic in both hands in five children (29.4%), and unilateral clonic movement in three children (17.6%). Nearly half the children with nonepileptic seizures (47%) were reported to show lower limb movements; there was asymmetrical clonic in five children (29.4%), unilateral clonic in two (11.8%), and symmetrical clonic in one child. No limb movements were reported in nine children (52.9%). Vocalization at the beginning or in the middle of the seizures was observed in four children (23.5%). Pelvic thrusting was reported in nearly one-fourth of the children (23.5%). Nearly one-third (35.3%) of the children with pseudo seizures were reported to experience whole body rigidity; whole body flaccidity was reported in only two children (11.8%). None of the nonepileptic seizure children was reported to experience any automatism, drooling of saliva, frothing, tongue bite, or fecal or urinary incontinence during the seizure like episodes. Unresponsiveness was fairly common and a majority (70.6%) of the group was found to experience it. Except one, all the children who were unresponsive were reported to be unconscious. Four children were observed to experience a seizure on suggestion and suppression of seizure on command, in the clinic. Out of the 17 children with nonepileptic seizures, the diagnosis was confirmed by video EEG in the case of twelve.

### Comparison of the groups on number of life events and stress scores

The mean scores on LESIC and CPMS for the three groups of children by sex are presented in Table 1. Comparison of the three groups on number of life events and associated stress scores for the preceding year and the year before indicated that the nonepileptic seizure group had significantly higher number of life events and stress scores in the preceding year as compared to the seizure group and healthy control group. However, there were no significant group differences in the mean number of life events and associated stress scores in the year before the preceding year. Some of the common stressors identified in the children with nonepileptic seizures included school examinations, beginning of a new school year, increase in the number of arguments between the parents, physical punishment by parents or school teacher, death of a family member, and failure in school.

**Table 1:** Mean scores on LESIC and CPMS for the three groups by sex

	Male	Female	Male	Female	Male	Female
No. of life events in preceding year	5.5	5.23	2	4.08	3.5	3.85
Stress scores in the preceding year	222	212.23	66	163.46	135.25	144.08
No. of events in the year before	.25	.54	.50	.85	.25	1.23
Stress scores in the year before	12	29.85	20.50	41.08	12.25	52.54
CPMS	10.75	13.62	10.5	10.85	17.75	7.77

Male: N=4, Female: N=13

In addition, there was a clear temporal relationship with the stressor and the seizure like episode in 11 (64.7%) children with nonepileptic seizures.

### Childhood psychopathology

Emotional and behavioral functioning at home was measured by the CPMS. Results indicate that there were no significant differences among the three groups in the mean CPMS score. However, significantly higher proportion of the nonepileptic seizure group (64.7%, N=11) and seizure group (52.9%, N=9) as compared to the control group (23.5%, N=4) were found to have CPMS scores above the cut off score and hence functioning in the clinically significant maladjustment range.

### Short-term outcome

All children with nonepileptic seizures had a follow-up for three to six months. The treatment and management of these generally followed the approach highlighted by previous studies. This involved (i) shifting the focus of the parents from an organic to a psychosocial explanation of the symptoms; (ii) encouraging the child and parents to resume normal activities; (iii) ignoring or discouraging sick role behavior; and (iv) using problem solving coping techniques to tackle the child's difficulties, and (v) family counseling for enhancing parental competence to tackle problems and resolving family crises.

Results indicated that six children (35.3%) were seizure free after three to six months, eight (47.1%) experienced more than 50% reduction in the frequency of the symptoms and three children (17.6%) were lost for follow-up. All children who showed improvement resumed normal activities, were attending school regularly and showed no instance of recurrence and there was no single instance of symptom substitution during the entire period of follow-up.

## Discussion

The present study examined the clinical features, psychosocial stressors and emotional and behavioral functioning of children with nonepileptic seizures. In keeping with findings from other studies, the majority of the pseudo seizure patients were female subjects.[10,13,14] Unresponsiveness without marked motor manifestations was the most common ictal characteristic of the nonepileptic seizures. Pelvic thrusting, upper and lower limb movement, head movements, vocalizations were observed in less than one-third of the patients. None of the nonepileptic seizure group of patients was reported to have fecal or urinary incontinence, automatisms, frothing, tongue bite or trauma during the episode. These findings support previous studies with adult subjects.[9,15] For example, Gulick *et al.*[15] found that 41% of patients with psychogenic seizures had episodes in which ictal features were impaired responsiveness with either complex behavior simulating epileptic automatisms or no significant motor behavior.

Increased psychosocial stress and higher number of life events in the preceding year were found to characterize children with nonepileptic seizures as compared to seizure group and control group. In addition, a clear temporal relationship between stressor and symptoms was reported for majority of the patients with nonepileptic seizures. Interestingly, the relationship between stressors and onset of symptoms was rarely apparent during history taking and a detailed interview was required in almost all cases to uncover the major etiological antecedent events. Previous authors have also stressed the importance of psychological stress as an etiological factor in children with conversion reactions including psychogenic seizures.[8,16–18] For example, Srinath *et al.*[8] reported significant stressors among 71% of children with conversion reactions, in a study from South India. Some of the stressors reported included punitive parenting, parental discord, sibling rivalry, academic difficulties and adjustment problems with peers. Wyllie *et al.*[10] found severe environmental stress, especially sexual abuse common among children and adolescents with nonepileptic seizures. In the present study, there was only one child in which the possibility of sexual abuse was considered, although it could not be confirmed.

A role model for the symptoms was reported in only one-third of the children with nonepileptic seizures. This may be due to the difficulty in eliciting the same as Taylor[19] has argued that the role model may not be current and may arise from the long forgotten past, real or imagined, and may also evolve during the course of the illness. Previous studies have also documented that a role model for the symptoms may not always be present.[18]

Children with nonepileptic seizures and true seizures were found to be at a higher risk for emotional and behavioral problems. The groups also had a higher proportion of children with psychopathology scores in the clinically significant maladjustment range as compared to healthy normal controls. Previous authors have also stressed that children with nonepileptic seizures and epilepsy have higher prevalence of psychiatric disorders and psychosocial difficulties[10,21–23] Wyllie *et al.*,[10] for example, found that major mood disorders were present in nearly one-third of children and adolescents with pseudo seizures. In a study from India, Malhi and Singhi[23] reported significantly higher psychopathology scores in children with conversion disorder, as compared to healthy controls.

All children were managed by communicating to the families the diagnosis and an explanation of the role of stress and emotional factors in the cause of pseudo seizures, in an unambiguous manner. The aim of the treatment was to help families develop an understanding of the etiological factors triggering or maintaining the seizures. Direct management techniques such as a providing additional academic support to a child with academic problems, recommending change of school for a child with major adjustment problems at school, asking parents to reduce open marital conflict for a child who is facing considerable stress due to increase in parental conflict, were also used. Parents were also asked to reduce secondary gain, if any, and normalize the child's activities, if the child showed significant functional impairment.

A striking feature of the study was the rapidity with which the symptoms went away, once the diagnosis was made and parents counseled regarding the same. A majority of the patients with nonepileptic seizures recovered within three to six months. Symptoms resolution helped the families to accept the validity of the diagnosis. Previous studies too have documented favorable outcome in children with nonepileptic seizures.[13,23–26] Wyllie *et al.*[26] studied 18 patients with psychogenic seizures and documented that 78% were seizure free at an average follow-up time of 2.5 years after diagnosis. Irwin *et al.*[13] studied 35 children with nonepileptic seizures and reported that nearly two-thirds of these children were seizure free and an additional 23% had more than 50% reduction in the frequency of seizures, after a mean follow-up duration of four to six years.

The high rate of favorable outcome in children and adolescents contrast with a relatively more guarded outcome in adults.[27,28] Possibly, the good prognosis in children with nonepileptic seizures found in our study may be due to the external nature of the cause of seizures which once identified was amenable to prompt intervention. It also remains possible that children require simpler techniques to lower stress levels as contrasted to adults.

In conclusion, it may be said that the diagnostic challenges of nonepileptic seizures have diminished in the era of video-EEG monitoring; nevertheless, the challenges of management still persist. Recognizing that the seizures are psychogenic, along with identification of the antecedent psychosocial stressors and emotional and behavioral functioning, may shed light on the underlying mechanism of symptom evolution and short- and long-term outcome.

Nonepileptic seizures have a good prognosis provided the diagnosis is made early, stressors are identified and addressed, and psychosocial interventions are initiated.
